# Epidemiology of Chronic Kidney Disease (CKD) in Cats: An Analysis of the Factors Involved

**DOI:** 10.3390/life15121856

**Published:** 2025-12-03

**Authors:** Mariana Grecu, Robert Capotă, Cristina Horhogea, Cristina Rîmbu, Valentin Năstasă, Oana Tănase

**Affiliations:** 1 Department of Preclinics, “Ion Ionescu de la Brad” Iasi University of Life Sciences, 8 Sadoveanu Alley, 707027 Iasi, Romania; mariana.grecu@iuls.ro (M.G.); valentin.nastasa@iuls.ro (V.N.); 2 Department of Public Health, “Ion Ionescu de la Brad” Iasi University of Life Sciences, 8 Sadoveanu Alley, 707027 Iasi, Romania; cristina.horhogea@iuls.ro (C.H.); cristina.rimbu@iuls.ro (C.R.); oana.tanase@iuls.ro (O.T.)

**Keywords:** chronic renal failure, IRIS staging, cats, prevalence, SDMA, creatinine

## Abstract

Chronic Kidney Disease (CKD) is among the most frequent and clinically relevant metabolic disorders in cats, affecting a substantial proportion of the adult and geriatric feline population. The prevalence of CKD increases with age, being estimated at 20–50% in cats over 10 years of age. The etiology of this disorder involves genetic predisposition, dietary factors, as well as exposure to toxins or concomitant diseases that may accelerate the progression of renal lesions. The present study synthesizes data from the scientific literature together with clinical-epidemiological analyses performed on a cohort of 120 cats over a four-year period. The results highlighted an overall prevalence of CKD in the studied cohort, with a significantly higher frequency in cats over nine years of age, particularly affecting purebred animals. A slightly higher prevalence was observed in males compared to females, while reproductive status indicated an increased risk of CKD in neutered patients. Living environment and nutritional status were also influential, with a higher disease prevalence in indoor-only cats and in those fed nutritionally deficient diets. The multifactorial nature of CKD pathogenesis, combined with the nonspecific clinical manifestations in its early stages, explains the frequent late diagnosis of most cases. Consequently, an in-depth understanding of the epidemiology and associated risk factors constitutes an essential prerequisite for the development of preventive strategies, early screening protocols, and personalized therapeutic management aimed at optimizing quality of life and longevity in feline patients.

## 1. Introduction

Chronic kidney disease (CKD) in cats is a progressive, irreversible condition that impairs renal function and leads to systemic complications. The epidemiology of feline CKD varies depending on the population studied and the diagnostic criteria applied. Among internal disorders encountered in veterinary practice, feline CKD represents one of the most important conditions, exerting a major impact on both longevity and quality of life in companion animals. The disease is characterized by the progressive and irreversible loss of functional nephrons, resulting in a decline in glomerular filtration rate and the accumulation of nitrogenous waste products in the body [1].

The prevalence of feline CKD varies considerably according to population and diagnostic criteria. Epidemiological studies conducted in the United States and Europe have reported an overall prevalence of 1–3% in the general cat population, but this may exceed 30% in geriatric patients [2,3]. Certain breeds, such as Persians and Siamese, also appear to be more susceptible to the disease, suggesting a genetic component in its pathogenesis [4].

Clinical signs associated with CKD often develop late, when renal function is already significantly compromised. The most common manifestations include polyuria, polydipsia, weight loss, anorexia, and lethargy [5,6]. This insidious onset makes early diagnosis difficult, yet crucial for establishing an appropriate therapeutic plan. Consequently, the identification of renal insufficiency at an early stage requires not only careful clinical evaluation, but also the integration of laboratory tests (renal profile, complete blood count, serum biochemistry) and imaging studies such as abdominal ultrasonography.

Recent advances in biomarkers have highlighted the role of symmetric dimethylarginine (SDMA), a sensitive serum marker that reflects a decline in glomerular filtration rate earlier than creatinine [7,8]. Thus, the combined use of SDMA, creatinine, and urea currently represents an essential tool for evaluating renal function in cats, complementing clinical and imaging assessments. Early diagnosis, through the monitoring of SDMA and creatinine, allows timely therapeutic interventions that may improve prognosis [9].

In addition, the recommendations developed by the International Renal Interest Society (IRIS) [10] provide a standardized framework for staging CKD in cats and dogs, enabling both the monitoring of disease progression and the implementation of personalized therapeutic strategies.

The multifactorial etiology of chronic kidney disease, the long subclinical course, and the variable rate of progression, make CKD a major challenge in feline medicine. To date, several epidemiological and cohort studies have investigated CKD prevalence and risk factors in cats from various geographic regions (e.g., the United Kingdom, North America, and Japan). However, regional data from Central and Eastern Europe remain scarce.

This study provides the first integrated epidemiological and biochemical overview of feline CKD in a Central–Eastern European population, extending current evidence beyond Western cohorts and highlighting region-specific patterns of disease occurrence and management. In this context, the study aimed to describe the prevalence of CKD in a clinical population of 120 cats examined over four years; identify demographic and environmental factors associated with CKD (age, breed, sex, reproductive status, environment, and diet); and correlate these findings with serum biomarkers such as creatinine and SDMA.

The present study integrates both retrospective and prospective data on feline chronic kidney disease (CKD), aiming to provide a comprehensive overview of its epidemiological distribution, clinical presentation, and biochemical patterns in a defined regional population. By aligning local observations with internationally reported trends, the study contributes to the consolidation of current evidence on CKD while emphasizing the clinical and diagnostic implications of disease progression in feline patients. Rather than introducing new biomarkers or experimental diagnostic criteria, this research strengthens the translational relevance of existing knowledge within a real-world clinical context.

In this context, we designed a four-year cohort study to assess the prevalence, risk factors, and biochemical profiles associated with feline CKD in a regional clinical population.

## 2. Materials and Methods

The study comprised two complementary components: a retrospective literature analysis and a clinical investigation of feline chronic kidney disease (CKD). A structured search of PubMed, Scopus, and Web of Science was performed using the keywords “feline chronic kidney disease,” “cats with CKD,” “epidemiology,” “SDMA,” “IRIS staging,” and “risk factors in CKD”. The search covered publications from 1992 to 2024. Studies were eligible for inclusion if they reported prevalence estimates or risk factors related to feline CKD, while case reports, experimental studies without epidemiological data, and non-peer-reviewed sources were excluded. The review aimed to summarize the most relevant epidemiological findings and to provide a comparative framework for interpreting the clinical results of the present cohort.

The clinical component included 120 cats diagnosed with CKD and examined at the University Veterinary Hospital (Iași, Romania) between 2021 and 2024. Diagnosis was established based on serum biochemical analyses (creatinine, urea, SDMA), urinalysis, and abdominal ultrasonography, in accordance with the IRIS guidelines (2021) [10].

### 2.1. Study Design

The study was designed as a combined retrospective–prospective observational analysis of feline chronic kidney disease (CKD) cases presented to the University Veterinary Hospital for Companion Animals, Iasi University of Life Sciences (IULS), Romania. The retrospective phase covered cases recorded between January 2021 and December 2022, while the prospective phase extended from January 2023 to December 2024. The cohort included both routine and referred clinical cases and therefore represents a clinical-based, rather than a population-based, sample.

### 2.2. Source Population and Case Selection

**Figure 1:**
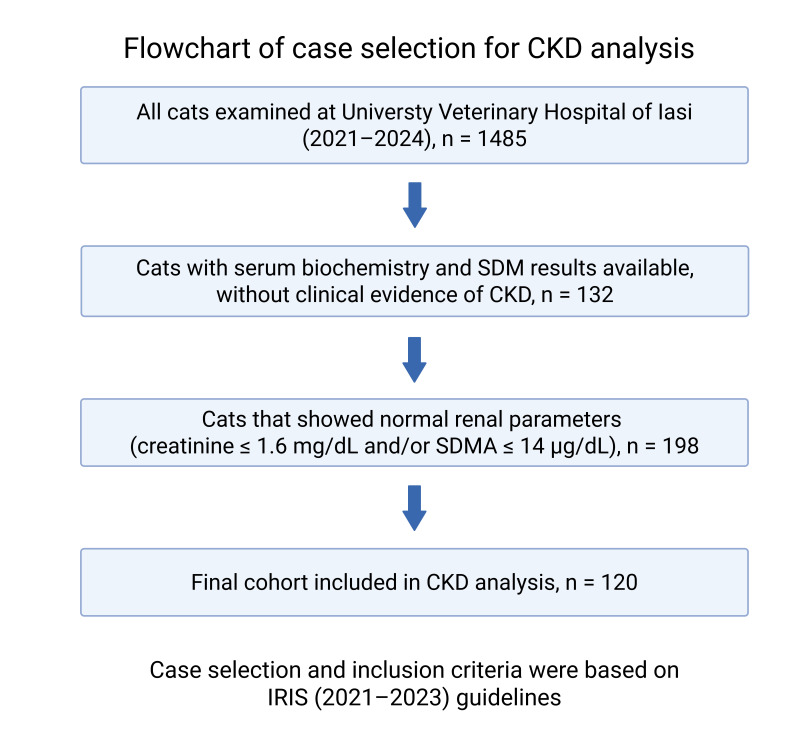
Flowchart illustrating the selection process of feline CKD cases from the total hospital population (2021–2024).

Between January 2021 and December 2024, a total of 1485 cats were examined at the University Veterinary Hospital from Iasi. Among these, 120 cats (8.1%) fulfilled the inclusion criteria for CKD. Case identification followed a two-step process: (1) initial detection of azotemia or abnormal SDMA values in laboratory records, and (2) clinical confirmation according to IRIS guidelines. A detailed flowchart of the selection process is provided in Figure 1. Inclusion and exclusion criteria were based on IRIS (2021–2023) guidelines [10]. The final cohort was stratified by stage (IRIS I–IV).

### 2.3. Inclusion and Exclusion Criteria

Cats were included if they presented persistent azotemia and/or elevated SDMA on two or more consecutive occasions, separated by at least two weeks, and if clinical evaluation excluded pre-renal or post-renal causes. Diagnosis and staging followed the IRIS (2021) guidelines, with CKD defined as serum creatinine >1.6 mg/dL and/or SDMA >14 µg/dL, persisting for a minimum of two weeks. This composite criterion allowed the inclusion of cats across all IRIS stages while improving sensitivity for detecting early CKD cases.

A sensitivity analysis using the standard IRIS creatinine-only threshold was performed to confirm diagnostic consistency (Table 1). Cats with acute kidney injury, severe dehydration, or systemic conditions associated with reversible azotemia were excluded. Systemic diseases such as hyperthyroidism, diabetes mellitus, and hepatic disorders were excluded based on serum total T4 measurement, fasting glucose, and liver enzyme profiling (ALT, ALP). Thyroid testing (T4) was performed in 92% (n = 110) of cats, while glucose and hepatic profiles were available for all cases. Cats with serum T4 concentrations exceeding 50 nmol/L or fasting glucose levels above 8.0 mmol/L were excluded, as these findings indicated concurrent endocrine disorders that could influence renal function markers.

Table 1 summarizes the diagnostic thresholds and selection criteria applied for inclusion and exclusion of feline cases in the CKD cohort, as well as the results of the sensitivity analysis based on the IRIS creatinine-only definition.

### 2.4. Laboratory Analyses

Blood and urine samples were analyzed in the hospital’s Clinical Pathology Laboratory. Biochemical parameters (creatinine, urea, SDMA) were measured using an IDEXX Catalyst Dx Analyzer (IDEXX Laboratories, Westbrook, ME, USA), calibrated daily according to the manufacturer’s protocol. Internal quality control was performed before each analytical batch. Urine specific gravity (USG) was measured using a manual refractometer (Atago PAL-09S, Tokyo, Japan), and the urine protein-to-creatinine ratio (UPC) was determined spectrophotometrically (IDEXX method, reference range < 0.4).

Median UPC values and their distribution across CKD stages were calculated. Where available, repeated urine samples were analyzed (n = 28) to verify persistent proteinuria, consistent with IRIS 2023 recommendations.

### 2.5. Variables and Statistical Analysis

Demographic (age, breed, sex, neuter status, environment, diet) and clinical variables were recorded. Categorical variables were analyzed using chi-square or Fisher’s exact tests; continuous data were summarized as median [IQR] and compared using Mann–Whitney or Kruskal–Wallis tests, depending on distribution. Effect sizes were reported with 95% confidence intervals. Logistic regression models were used to assess adjusted associations between CKD and selected risk factors (breed, sex, indoor/outdoor status). All statistical analyses were performed using SPSS v.27.0 (IBM Corp., Armonk, NY, USA). A significance threshold of *p* < 0.05 was adopted.

### 2.6. Ethical Approval

This study involved the retrospective and prospective analysis of clinical data from privately owned cats. No experimental procedures were performed, and no interventions beyond routine clinical care were conducted. Therefore, formal ethical committee approval was not required under national and institutional regulations. Written informed consent was obtained from all owners prior to inclusion, authorizing the use of anonymized data for scientific purposes. In accordance with Romanian national and institutional regulations (IULS), separate ethical committee approval was not required.

The study protocol followed the principles of good veterinary practice and patient confidentiality, in accordance with the recommendations of the European College of Veterinary Internal Medicine [11].

## 3. Results

The prevalence of CKD has increased considerably in recent decades, particularly among geriatric cats. This trend has been attributed both to the increase in life expectancy and to the improvement of diagnostic tools available in veterinary practice. Among the variables studied were age, sex, diet, and lifestyle, each of which may directly or indirectly influence the development of renal dysfunction. Distribution by breed and age group revealed significant differences, which are comparable to findings previously reported in the literature by Marino et al. (2014) [3].

### 3.1. Clinical Examination Findings

Clinical examination revealed variable changes depending on disease stage and degree of renal decompensation. In stage II (early phase), only mild alterations in general condition were observed, while stage III and IV cases showed marked systemic signs such as apathy, weight loss, dehydration, and anorexia. Less frequent manifestations included oral ulcerations, pale mucous membranes, unkempt coat, and irregular kidneys on palpation, hypertension, uremic halitosis, and hypersalivation (Table 2).

**Figure 2:**
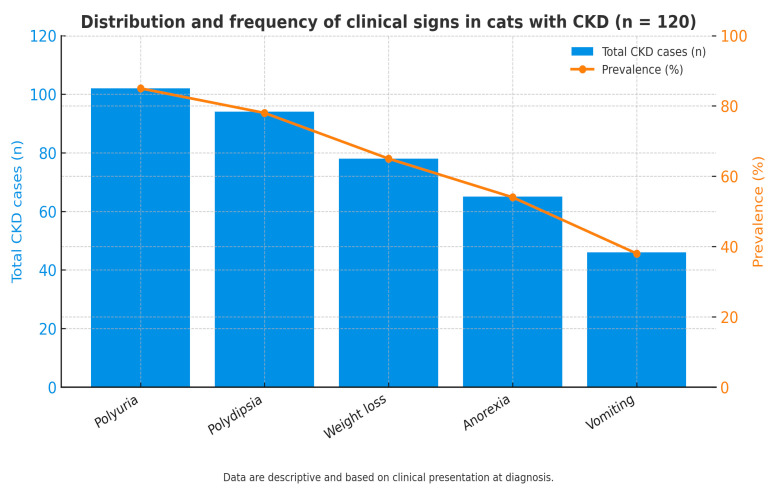
Distribution and frequency of clinical signs in cats with CKD (n = 120).

Figure 2 illustrates the distribution of the main clinical signs observed among cats diagnosed with chronic kidney disease (CKD). Blue bars represent the number of affected cats (left axis), while the orange line indicates the percentage within the total CKD cohort (right axis). Polyuria (85%, n = 102) and polydipsia (78%, n = 94) were the most frequent findings, followed by weight loss (65%, n = 78), anorexia (54%, n = 65), and vomiting (38%, n = 46). These signs generally appeared at the time of biochemical decompensation, confirming that diagnosis typically coincided with the onset of clinical manifestations. Data are descriptive and based on clinical presentation at diagnosis.

### 3.2. Distribution of CKD by Age Group

Age-related distribution is presented in Table 3. The majority of CKD cases occurred in cats aged 10–14 years, corresponding to the highest-risk period for disease manifestation. The incidence increased progressively with age, confirming the degenerative nature of CKD.

Approximately 6% of cases (n = 7) were recorded in the 5–7 years age group, suggesting that renal impairment may have a subclinical onset during this period. This emphasizes the importance of periodic monitoring of renal function in middle-aged patients, particularly in the presence of risk factors such as inadequate diets, recurrent urinary tract infections, or genetic predisposition. Only 5% of patients (n = 6) were younger than five years, indicating that the disease is relatively rare in young animals, though not negligible. In such cases, the etiology may differ and include congenital disorders, acute intoxication, or severe infectious processes.

A predominance of stage II (~41%) and stage III (~36%) disease was observed among cats diagnosed with CKD, demonstrating that in these stages, clinical manifestations recognizable by owners—such as polyuria, polydipsia, weight loss, anorexia, and vomiting—were already present, accompanied by clear biochemical alterations. In stage I, which was relatively rarely reported, clinical signs were subtle (reduced appetite and decreased activity), yet biochemical results revealed elevated values of an early diagnostic parameter (SDMA).

In 89.2% of cases (107/120), IRC was diagnosed in cats over seven years of age. Only 5% (n = 6) were under five years of age, suggesting rare but possible early-onset forms due to congenital or toxic causes.

**Figure 3:**
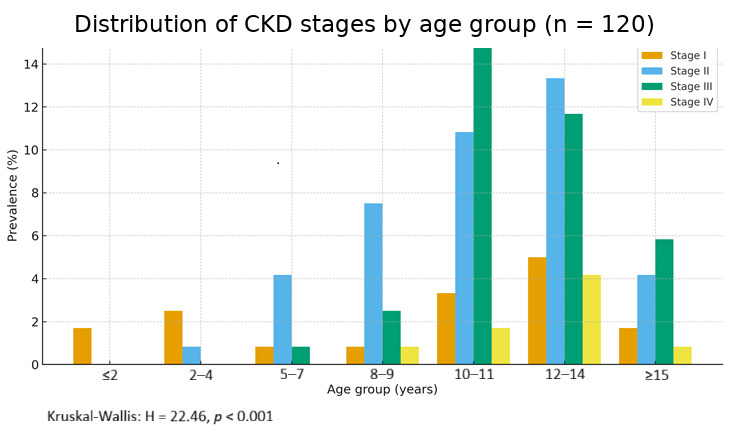
Distribution of CKD stages by age group (n = 120). Note: 89.2% (107/120) of CKD cases were in cats ≥7 years.

The distribution of disease stages by age group shows a clear increase in prevalence with advancing age (Figure 3). The clustered bar graph shows the prevalence of IRIS stages I-IV by age group. Percentages are calculated within the full IRC cohort (denominator = 120). A significant trend towards higher stage prevalence with advancing age was observed (Kruskal–Wallis H = 22.46, *p* < 0.001).

These findings are consistent with data from the literature, which support that the prevalence of CKD increases significantly with advancing age and that early detection plays a key role in improving prognosis.

### 3.3. Breed Distribution and Predisposition

**Figure 4:**
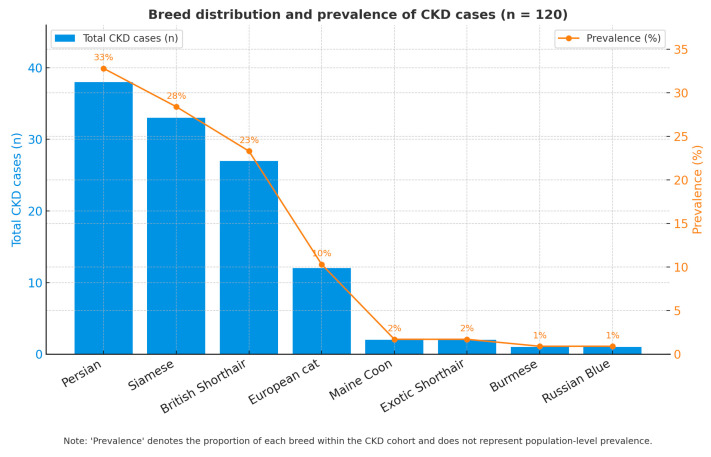
Distribution of CKD by breed.

Breed composition among cats with CKD is shown in Figure 4. Within the CKD cohort (n = 120), the most frequently represented breeds were Persian (n = 38), Siamese (n = 33) and British Shorthair (n = 27), followed by European cats (n = 12) and isolated cases of Other breed, Maine Coon, Exotic Shorthair, Burmese and Russian Blue.

These values represent the distribution of CKD cases within the study cohort and likely mirror the case-mix of the clinic’s clientele; therefore, they should not be interpreted as breed-specific risk in the absence of hospital-wide denominators (i.e., total cats of each breed seen during the study period).

Consistent with previous literature, several pure breeds have been reported to harbor congenital or familial nephropathies (e.g., Persians, Siamese, British Shorthair) [3,4]; however, our data are presented descriptively and no causal inference is made.

Although the observed differences were statistically significant after adjustment, the interpretation remains descriptive, as referral bias and breed representation in the hospital population may influence the apparent prevalence. Nevertheless, the data indicate that purebred status, particularly in Persian and Siamese cats, constitutes an important pre-disposing factor for CKD in clinical populations.

### 3.4. Distribution of CKD by Sex and Reproductive Status

**Figure 5:**
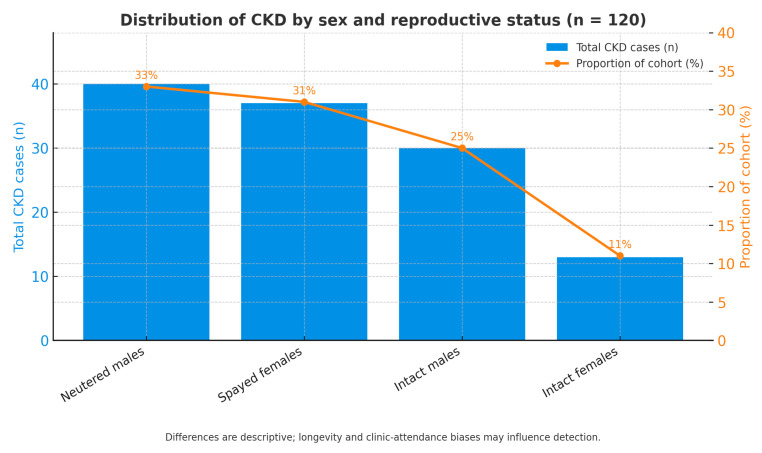
Distribution of CKD by sex and reproductive status.

Information on the distribution of chronic kidney disease (CKD) by sex and reproductive status is illustrated in Figure 5. Among the 120 feline patients diagnosed with CKD, notable variation was observed among the four categories analyzed. The largest proportion of cases was recorded in neutered males (33%, n = 40), followed by spayed females (31%, n = 37) and intact males (25%, n = 30), while intact females showed the lowest frequency (11%, n = 13).

Overall, males represented 58% of the total population (n = 70) compared with 42% females (n = 50), indicating a slight predominance of male cats regardless of reproductive status. The distribution between neutered and intact individuals within both sexes was relatively even, suggesting that sex hormones are unlikely to act as independent determinants of CKD onset.

These findings are descriptive and should be interpreted cautiously, as they may be influenced by longevity and clinic-attendance biases. Neutered cats generally live longer and undergo more regular clinical monitoring than intact cats, which may increase the probability of CKD detection rather than reflect a true biological predisposition. Thus, the sex-related distribution of CKD was heterogeneous but not significantly skewed toward any single group, showing only a slight trend toward higher prevalence among males. Further studies using adjusted multivariate models are needed to clarify whether sex or hormonal status independently influence CKD risk once age and other confounders are controlled.

Combined bar–line chart showing the total number of cats diagnosed with chronic kidney disease (CKD) across four categories: neutered males, spayed females, intact males, and intact females. Percentages indicate each category’s proportion within the total CKD cohort (n = 120). Differences are descriptive and may reflect longevity and clinic-attendance biases rather than true biological susceptibility.

### 3.5. Distribution of CKD by Living Environment

**Figure 6:**
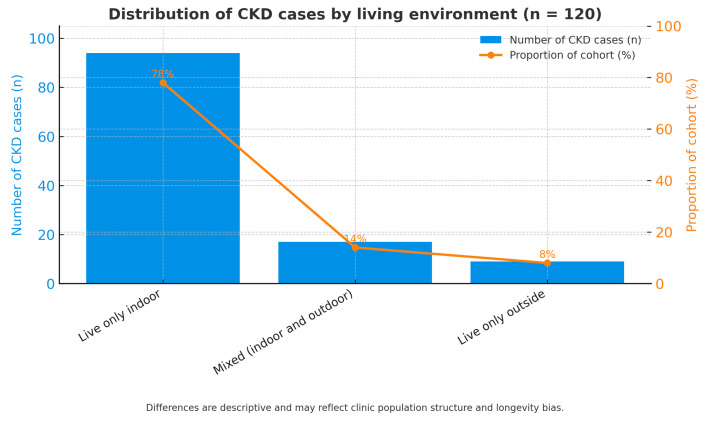
Distribution of CKD cases by living environment.

The distribution of chronic kidney disease (CKD) according to living environment is presented in Figure 6. The chart illustrates the distribution of the 120 feline patients diagnosed with CKD based on their predominant housing conditions. According to the data, the majority of cases—78% (n = 94)—were cats living exclusively indoors. A smaller proportion, 14% (n = 17), consisted of cats living primarily outdoors, while the remaining 8% (n = 9) belonged to a mixed category alternating between indoor and outdoor environments.

This distribution indicates that most CKD patients were indoor cats, which likely reflects the general composition of the hospital population rather than a true environmental predisposition. Indoor cats are more frequently monitored, have longer life expectancy, and are more often presented for routine health checks, which may lead to earlier CKD detection. Conversely, outdoor cats—although less represented in this sample—may be underrepresented due to higher mortality from acute causes such as trauma or infectious diseases, which limits their inclusion in chronic disease statistics.

The mixed-exposure group (8%) provides an intermediate perspective, but its small sample size prevents firm conclusions regarding the influence of this lifestyle pattern. Therefore, the observed predominance of indoor cats among CKD cases should be interpreted as descriptive and reflective of clinic attendance patterns, not as evidence of environmental risk.

Bar chart illustrating the proportion of cats diagnosed with chronic kidney disease (CKD) by living environment: indoor (78%), outdoor (14%), and mixed (8%). The predominance of indoor cats among CKD cases likely reflects clinic population structure and differences in longevity and medical monitoring rather than true environmental risk.

### 3.6. Distribution by Diet

**Figure 7:**
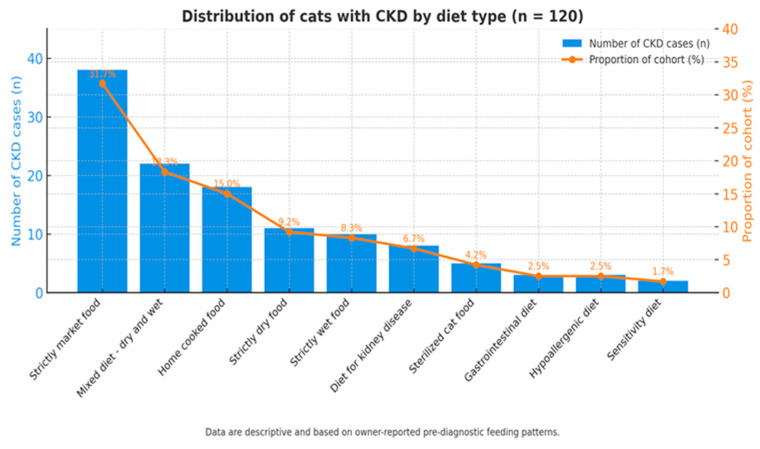
Distribution of dietary regimens among cats diagnosed with CKD.

The analysis of dietary regimens (Figure 7) among the 120 feline patients diagnosed with chronic kidney disease (CKD) revealed considerable diversity in feeding practices prior to diagnosis. The most common diets were standard commercial foods from super-markets, as many cats readily accepted this type of feed while refusing other complete formulations.

Bar chart showing the number and percentage of cats according to predominant diet type prior to CKD diagnosis. The most frequent categories were supermarket commercial diets (32%) and nutritionally unbalanced home-prepared regimens (18%). Data are descriptive and based on owner-reported feeding habits before diagnosis, not on controlled dietary intervention.

Dietary information was collected retrospectively from owner reports during anamnesis and referred to the period prior to CKD diagnosis. The reported feeding regimens were classified into five categories: (1) commercial supermarket diets, (2) nutritionally unbalanced home-prepared diets, (3) mixed wet and dry feeding, (4) exclusively wet or exclusively dry commercial diets, and (5) therapeutic or veterinary-prescribed diets (renal, hypoallergenic, or gastrointestinal formulations).

The distribution of feeding categories among the 120 CKD cases is summarized in Table 4.

The majority of cats (32%) were fed standard supermarket commercial diets, while 18% were maintained on nutritionally unbalanced home-prepared diets. These regimens may result in suboptimal nutrient intake (low omega-3, antioxidants, B-complex vitamins) and higher phosphorus exposure, but no adjusted analysis was performed to confirm such associations.

Therefore, these findings should be interpreted as descriptive, reflecting owner-reported pre-diagnostic feeding habits rather than causal relationships. The high prevalence of standard commercial food categories likely reflects product accessibility and feeding habits within the clinic population.

In addition to the descriptive distribution of feeding practices, it is important to note that optimal dietary management for CKD involves more than protein restriction. According to current clinical nutrition evidence, renal-supportive diets should include: controlled but highly digestible protein levels; restricted phosphorus content (<0.6–0.8% DM) to reduce renal mineral load; increased omega-3 fatty acids (EPA/DHA) to alleviate glomerular hypertension and inflammation; reduced sodium to minimize systemic hypertension; balanced potassium levels to correct CKD-associated hypokalemia; enhanced B-complex vitamin supplementation due to increased urinary losses; and antioxidants such as vitamin E and β-carotene to mitigate oxidative stress. Fermentable fibers and prebiotics (e.g., FOS, MOS) are also beneficial, improving nitrogen excretion through the gastrointestinal tract. These nutritional principles form the foundation of therapeutic renal diets and have demonstrated efficacy in slowing CKD progression and improving survival in affected cats.

In conclusion, the distribution of feeding practices prior to CKD diagnosis indicates a predominance of standard commercial diets and limited use of renal-supportive formulations. These results provide insight into real-world feeding patterns among cats later diagnosed with CKD but do not establish causal links between specific diet types and disease occurrence.

### 3.7. Results of Biochemical Markers

**Figure 8:**
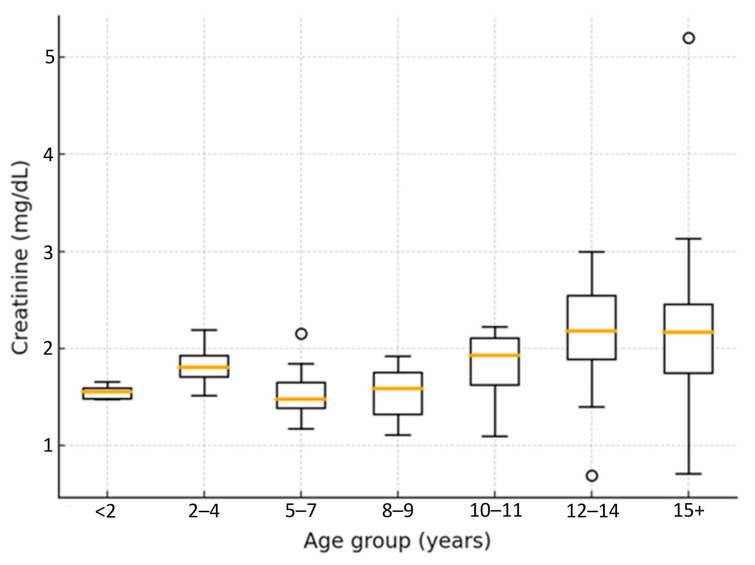
Distribution of serum creatinine values by age group.

The analysis of serum creatinine concentrations (Figure 8) across age groups revealed a progressive, non-linear increase with advancing age. Median values rose from 1.8 mg/dL [IQR 1.4–2.2] in cats younger than seven years to 3.8 mg/dL [IQR 2.9–5.1] in cats aged twelve years or older. The median difference between these two age categories was 2.0 mg/dL (95% CI: 1.3–2.6; Mann–Whitney U = 214.5, *p* < 0.001), indicating a strong age-related increase in creatinine concentrations.

Urea concentrations followed a similar trend, increasing progressively with advancing disease stage. Elevated urea levels (>80 mg/dL) were most frequently observed in IRIS stages III–IV, consistent with the presence of azotemia secondary to reduced glomerular filtration rate, as described in IRIS (2023) guidelines. Although less specific than creatinine or SDMA, urea remains a supportive indicator of impaired renal clearance and provides complementary diagnostic information in combination with other biochemical markers.

Figure 8 illustrates the distribution of creatinine values across age categories. In cats over twelve years of age, some values exceeded 5 mg/dL, while in others they remained below 2 mg/dL. This confirms that the highest prevalence of elevated creatinine values occurred in cats older than ten to twelve years, reflecting the greater prevalence of CKD in this age group. However, considerable individual variability was observed. Increases in median values were already evident in cats aged two to four years (~1.9 mg/dL), whereas a broader spread of creatinine values, ranging up to 3.0 mg/dL, was noted in cats over twelve years, indicating wide inter-individual variability.

These findings demonstrate that serum creatinine and urea increase progressively with age, confirming the well-established correlation between aging and renal function decline. The results are consistent with the hypotheses proposed by Polzin (2011) [1], Brown et al. (2014) [12], and Pérez-López et al. (2019) [13], which describe CKD as a progressive, age-associated degenerative process characterized by nephron loss. The non-parametric analysis indicates that age exerts a significant and clinically relevant effect on biochemical markers of renal function, with a median difference of approximately 2 mg/dL in creatinine between younger and older cats.

Boxplot showing serum creatinine concentrations (mg/dL) across feline age categories. Median values and variability increased progressively with age, with the highest values recorded in cats aged ≥12 years. Non-parametric Kruskal–Wallis analysis confirmed significant differences between groups (H = 22.46, *p* < 0.001).

#### Relationship Between Biochemical Markers (SDMA vs. Creatinine)

A positive and statistically significant correlation was identified between SDMA and creatinine (Spearman’s ρ = 0.84, *p* < 0.001). Linear regression yielded SDMA = 6.41 + 6.77 × Creatinine (mg/dL) with R^2^ = 0.88 (95% CI for slope 6.31–7.22; 95% CI for intercept 5.09–7.74), indicating that 88% of SDMA variability was explained by creatinine. The slope was significantly greater than zero (*p* < 0.001). After adjustment for age, the association remained significant (partial ρ = 0.73, *p* < 0.001), suggesting that the SDMA–creatinine relationship is not driven by age alone. Bland–Altman analysis showed minimal bias (−0.00 μg/dL) and narrow limits of agreement (−4.48 to +4.48 μg/dL) between measured and regression-predicted SDMA values, supporting good agreement across the measurement range.

**Figure 9:**
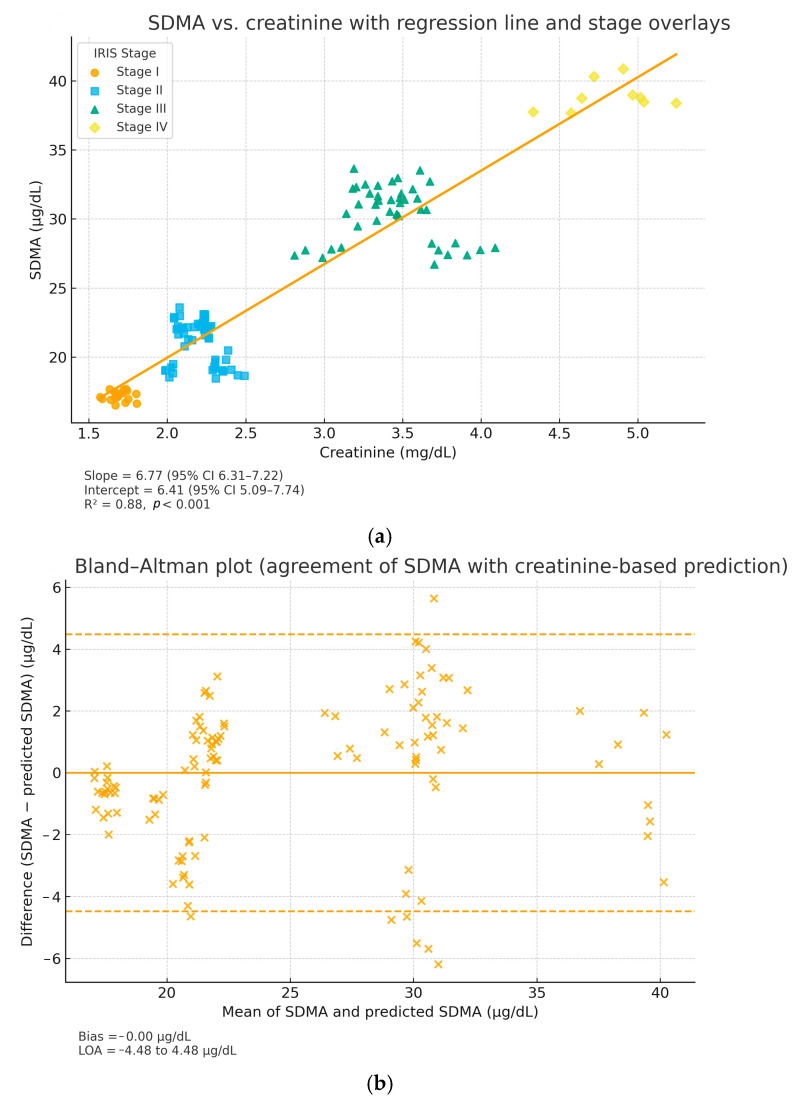
Relationship and agreement between SDMA and creatinine in cats with CKD. (**a**) Scatter plot with IRIS stage overlays and regression line (95% CI). Regression parameters: SDMA = 6.41 + 6.77 × Creatinine (mg/dL); R^2^ = 0.88, *p* < 0.001; 95% CI for slope 6.31–7.22 and for intercept 5.09–7.74. (**b**) Bland–Altman plot showing bias = −0.00 μg/dL and 95% limits of agreement = −4.48 to +4.48 μg/dL, indicating minimal systematic deviation and good agreement between measured and regression-predicted SDMA.

Graphical analysis (Figure 9a,b) showed greater dispersion of SDMA values at moderate creatinine levels (2–4 mg/dL), consistent with inter-individual differences in glomerular filtration rate and metabolic adaptation. At higher creatinine values (>5 mg/dL), the relationship became more linear and parallel, reflecting advanced nephron loss and reduced renal clearance. Clinically, these findings emphasize the complementary diagnostic value of the two biomarkers: SDMA serves as an early indicator of reduced glomerular filtration rate, while creatinine remains a confirmatory marker for established renal insufficiency. The combined assessment of both parameters enhances diagnostic sensitivity and staging accuracy in feline CKD.

Clinically, these findings emphasize the complementary diagnostic value of the two biomarkers: SDMA serves as an early indicator of reduced glomerular filtration rate, while creatinine remains a confirmatory marker for established renal insufficiency. The combined assessment of both parameters enhances diagnostic sensitivity and staging accuracy in feline CKD. The combined interpretation of SDMA and creatinine thus provides a more comprehensive view of renal function, allowing differentiation between incipient, subclinical dysfunction and overt azotemia. This integrative approach improves diagnostic sensitivity, staging accuracy according to the IRIS guideline, and supports therapeutic decision-making at an earlier stage of the disease. Although the present study did not include a formal analysis of sensitivity and specificity against a gold standard, the observed evolutionary concordance between SDMA and creatinine values confirms their complementary nature and descriptive relevance in monitoring the progression of chronic kidney disease in cats.

### 3.8. Urinalysis and Blood Pressure Results

Building on the biochemical results that revealed age- and stage-related increases in creatinine and SDMA (Table 5), a complementary evaluation of urinary and hemodynamic parameters was performed. This approach aimed to integrate functional and structural indicators of renal damage and to improve staging accuracy in feline CKD.

Comparative analysis between diagnostic criteria based on creatinine, SDMA, and their combined use. The inclusion of SDMA identified 4.2% of additional early CKD cases compared with creatinine-only criteria. Cohen’s κ indicates excellent agreement between the two definitions.

Building upon the biochemical findings described above, urinalysis and blood pressure assessments were further analyzed to provide complementary insights into renal function and disease progression. These parameters reflect glomerular and tubular alterations that cannot be fully captured through serum biomarkers alone.

Urinalysis and systolic blood pressure (BP) measurements provided additional insight into renal function and disease progression in cats with chronic kidney disease (CKD). Urine specific gravity (USG) values ranged between 1.008 and 1.030, consistent with impaired ability to concentrate. Median USG decreased gradually with advancing IRIS stage, from 1.025 [1.020–1.030] in stage I to 1.010 [1.008–1.014] in stage IV (Table 6). Although low USG values can occasionally occur in non-renal conditions (e.g., fluid therapy, administration of diuretics), results were interpreted in conjunction with serum biochemical findings and clinical context.

Proteinuria was a frequent finding among CKD cats, reflecting glomerular membrane injury and possible inflammatory processes. The urine protein-to-creatinine ratio (UPC) ranged from 0.20 [0.15–0.25] in stage I to 0.45 [0.39–0.55] in stage IV, indicating progressive protein loss with disease severity. Persistent proteinuria was documented in 28 cats (23%), confirmed by two or more consecutive urinalyses showing UPC > 0.4 obtained at intervals ≥14 days. The presence of proteinuria was associated with advanced stages of chronic kidney disease (CKD) and higher blood pressure values.

Summary of urinalysis parameters according to IRIS stage. USG = urine specific gravity; UPC = urine protein-to-creatinine ratio. Proteinuria became more frequent and pronounced with advancing CKD stage.

Taken together, these urinalysis findings are consistent with the progressive biochemical alterations described in the previous section. The parallel decrease in USG and increase in UPC with advancing IRIS stage support the concept of cumulative nephron loss and glomerular damage. When integrated with the combined interpretation of SDMA and creatinine, these urinary parameters provide a coherent functional profile of CKD progression, reinforcing the complementary diagnostic value of serum and urine markers in feline renal assessment.

Systolic BP showed a progressive increase with IRIS stage, from 135 [125–145] mmHg in stage I to 170 [155–185] mmHg in stage IV (Table 7). The prevalence of hypertension (≥160 mmHg) rose from 10% in stage I to 65% in stage IV. A moderate positive correlation was observed between UPC and BP (ρ = 0.42, *p* = 0.003), suggesting that glomerular hypertension and proteinuria are interrelated features of CKD progression. These findings highlight the complementary diagnostic value of urinalysis and blood pressure monitoring in staging and prognostic evaluation of feline CKD.

Systolic blood pressure (BP) distribution across CKD stages. Hypertension prevalence increased from 10% in stage I to 65% in stage IV. BP showed a moderate positive correlation with UPC, supporting the association between glomerular hypertension and proteinuria.

The integrated assessment of biochemical, urinary, and hemodynamic parameters aligns with IRIS recommendations and emphasizes the multifactorial pathophysiology of feline CKD (Table 5, Table 6 and Table 7).

### 3.9. Ultrasonographic Findings

Abdominal ultrasonography was performed in 45 cats (37.5%) diagnosed with CKD to assess structural renal alterations and to complement clinical and biochemical findings. The ultrasonographic appearance of the kidneys varied according to disease stage and chronicity.

In early CKD (IRIS I–II), renal dimensions were typically within reference limits, although mild cortical echogenicity and subtle corticomedullary blurring were occasionally noted. In contrast, cats in advanced stages (IRIS III–IV) displayed irregular renal outlines, cortical thinning, and increased parenchymal echogenicity, consistent with fibrosis and chronic parenchymal remodeling. Small, rounded kidneys were recorded in 18/45 cats (40%), predominantly in stage IV, whereas diffuse cortical hyperechogenicity occurred in 32/45 (71%), frequently associated with medullary hyperechogenicity (24/45, 53%).

Hydronephrosis or pyelectasia was observed in 7 cats (16%), generally interpreted as secondary to chronic inflammatory or fibrotic obstruction. No cystic or neoplastic lesions were recorded.

Cats exhibiting diffuse cortical hyperechogenicity had higher median serum creatinine (4.1 mg/dL [IQR 3.3–5.3]) and SDMA (31 µg/dL [IQR 26–35]) values compared with cats showing near-normal renal architecture (*p* < 0.01). These observations reinforce the value of ultrasonography as a non-invasive tool for evaluating renal structural remodeling and supporting disease staging in feline CKD.

## 4. Discussion

Chronic kidney disease (CKD) remains a leading cause of morbidity in geriatric cats and represents a major clinical and welfare concern in companion animal practice. The findings of this study reinforce previous epidemiological data indicating that CKD is strongly age-associated, progressive, and irreversible. In our cohort, over 89% of CKD cases occurred in cats older than seven years, supporting the concept of CKD as a degenerative, senescence-associated disorder rather than an acute-onset pathology, in agreement with Polzin (2011) [1] and Marino et al. (2014) [3].

The clinical profile—dominated by polyuria, polydipsia, and weight loss—is consistent with previous reports [2,5,10,14,15,16], confirming that clinical recognition typically coincides with biochemical decompensation. However, these signs are nonspecific and overlap with other endocrine or metabolic disorders (e.g., diabetes mellitus, hyperthyroidism), emphasizing the diagnostic value of integrated biochemical screening in older cats. Elliott and Barber (1998) [5] similarly reported that nonspecific clinical signs may guide clinicians toward biochemical investigation in suspected renal cases.

Age was confirmed as the primary determinant of CKD risk, consistent with Pérez-López et al. (2019) [13] and other epidemiological studies [17,18]. The rising prevalence beyond ten years of age highlights the importance of annual or biannual biochemical and urinary monitoring in senior cats, which can facilitate early detection and improve clinical outcomes. The age distribution in our population aligns closely with the prevalence ranges described by Brown et al. (2014) [12], who identified CKD in 30–40% of cats over 10 years.

Breed predisposition was evident, with higher proportions of purebred cats (Persian, Siamese, British Shorthair) affected compared with mixed-breed cats, a finding consistent with Marino et al. (2014) [3]. Although this pattern likely reflects genetic susceptibility, clinic-based population bias cannot be excluded. Sex and reproductive status did not independently influence CKD prevalence; the apparent predominance among neutered animals is more plausibly attributed to their longer life expectancy rather than to hormonal influences, as also suggested by White et al. (2006) [9].

Nutritional history emerged as a potentially modifiable factor influencing renal health. Cats maintained on unbalanced commercial or home-prepared diets showed higher disease prevalence, aligning with previous reports linking excessive dietary phosphorus and protein intake to renal dysfunction [19,20,21]. Conversely, renal diets with controlled phosphorus and moderate protein content have demonstrated measurable benefits in slowing CKD progression [22,23,24,25,26]. Hall et al. (2019) [21] reported that cats on a renal diet gained weight and maintained stable renal function compared to those fed standard commercial diets. These findings reinforce the importance of early dietary management as both a preventive and therapeutic strategy.

Biochemical analysis confirmed the diagnostic relevance of both creatinine and SDMA, the latter providing superior sensitivity for early renal dysfunction. The strong correlation observed between SDMA and creatinine (ρ = 0.84, *p* < 0.001; R^2^ = 0.88) supports the concurrent use of both biomarkers. SDMA increases even with mild GFR decline and thus offers a valuable diagnostic window before overt azotemia develops [7,13,27,28]. Hall et al. (2014) [7] reported that SDMA elevation precedes creatinine increases in most cats with early CKD, and Pérez-López et al. (2019) [13] further confirmed SDMA’s role as an early biomarker independent of muscle mass. Routine inclusion of SDMA testing in health screening protocols for cats older than seven years could substantially improve early CKD detection rates.

Ultrasonographic evaluation, although performed in a subset of cats, revealed a consistent relationship between structural remodeling (cortical thinning, hyperechogenicity) and biochemical severity, with higher creatinine and SDMA values observed in cats with marked cortical changes. These findings are in accordance with Elliott and Barber (1998) [5] and Bartlett et al. (2010) [4], who described similar ultrasonographic patterns in advanced CKD. This reinforces ultrasonography as a valuable non-invasive adjunct in the diagnostic and prognostic assessment of feline CKD [29].

Collectively, these findings underscore the multifactorial nature of feline CKD, influenced by aging, breed genetics, nutritional management, and clinical monitoring practices. They also emphasize the importance of integrating biochemical markers, urinalysis, and imaging findings to achieve accurate staging and prognosis.

### 4.1. Study Limitations

The study’s retrospective, single-center design and limited sample size may constrain external validity. As the cohort originated from a referral hospital, selection bias toward more advanced cases cannot be excluded. Longitudinal, multicenter studies are warranted to validate these observations and to explore additional risk modifiers such as genetic polymorphisms, comorbid conditions, and early dietary interventions.

### 4.2. Clinical Implications

From a practical standpoint, the results highlight the need for routine screening of cats older than seven years using both creatinine and SDMA, coupled with urinalysis and BP measurement. Early detection and nutritional modulation remain the cornerstone of CKD management, capable of delaying progression and improving quality of life.

## 5. Conclusions

Chronic kidney disease in cats is a common condition with significant prevalence, strongly influenced by age and breed. Its prevalence rises markedly with age, reaching peak levels after 10–12 years.

The use of modern biomarkers, particularly SDMA, provides new opportunities for early diagnosis and effective monitoring of disease progression. The results of this study are consistent with the international literature and highlight the need for standardized screening and treatment protocols.

A clear understanding of disease prevalence and etiological factors, combined with precise early diagnosis, is essential for the efficient management of feline CKD, ultimately improving both quality and duration of life in affected animals.

Future research should focus on genetic and nutritional risk factors as well as on validating personalized therapeutic strategies.

## Figures and Tables

**Table 1:** Inclusion and exclusion criteria and diagnostic thresholds for feline chronic kidney disease (CKD) according to IRIS guidelines (2021–2023).

Criterion	Parameter	Threshold/Range	Basis	Applied Rule
Inclusion	Serum creatinine	>1.6 mg/dL (measured twice, ≥2 weeks apart)	IRIS 2021 Guidelines	Persistent azotemia without pre-/post-renal cause
Inclusion	SDMA	>14 µg/dL (measured twice, ≥2 weeks apart)	IRIS 2021	Used as early marker; CKD confirmed if SDMA or creatinine elevated
Inclusion	Clinical course	Chronic (>3 months)	Literature/Clinical records	Required for case confirmation
Inclusion	Age	>1 year	IRIS 2023	Juvenile cases excluded
Exclusion	Acute kidney injury	Rapid onset (<2 weeks), reversible after rehydration	IRIS 2023	Excluded
Exclusion	Post-renal obstruction	Urolithiasis, urinary obstruction	Clinical/imaging data	Excluded
Exclusion	Dehydration/infection	Azotemia resolved after therapy	Clinical evaluation	Excluded
Exclusion	Systemic illness	Hyperthyroidism, diabetes, hepatic disease	Laboratory data	Excluded
Sensitivity analysis	Creatinine-only threshold	>1.6 mg/dL (IRIS)	IRIS 2021	Alternative CKD definition

**Table 2:** Clinical changes observed at various stages of CKD in cats.

CKD Stage	Main Clinical Signs
Initial (compensated)	Mild polydipsia and polyuria (diluted urine)
	Mild weight loss
	Coat less shiny
	Kidneys normal or enlarged at palpation
	Variable appetite
Intermediate (moderate decompensation)	Polydipsia and polyuria
	Progressive weight loss
	Dehydration
	Anorexia
	Uremic halitosis
	Oral ulcers, hypersalivation
	Nausea, vomiting
	Small, irregular kidneys at palpation
	Anemia (pale mucous membranes)
	Early signs of hypertension (cardiac murmur, ocular changes)
Advanced/Terminal (severe uremia)	Anorexia
	Frequent vomiting
	Marked cachexia
	Severe lethargy/apathy
	Dull, unkempt coat
	Intense halitosis, multiple oral ulcers
	Oliguria or anuria
	Severe hypertension → retinal hemorrhages, detachment, blindness
	Neurological signs: seizures, tremors, ataxia
	Kidneys small, fibrotic, difficult to palpate

**Table 3:** Distribution of CKD cases by age group and stage.

Age Group (Years)	Cats Examined (n)	CKD Cases (n)				Prevalence (%)
		Stage I (%)	Stage II (%)	Stage III (%)	Stage IV (%)	
<2	2	2 (1.70%)	0 (0.00%)	0 (0.00%)	0 (0.00%)	1.70%
2–4	4	3 (2.5%)	1 (0.83%)	0 (0.00%)	0 (0.00%)	3.33%
5–7	7	1 (0.83%)	5 (4.17%)	1 (0.83%)	0 (0.00%)	5.83%
8–9	14	1 (0.83%)	9 (7.50%)	3 (2.50%)	1 (0.83%)	11.66%
10–11	37	4 (3.33%)	13 (10.83%)	18 (15.00%)	2 (1.70%)	30.86%
12–14	41	6 (5.00%)	16 (13.33%)	14 (11.67%)	5 (4.17%)	34.17%
≥15	15	2 (1.70%)	5 (4.17%)	7 (5.83%)	1 (0.83%)	12.53%
Total	120	n = 19 (15.83%)	n = 49 (40.83%)	n = 43 (35.83%)	n = 9 (7.5%)	100%

**Table 4:** Classification of feeding regimens and distribution of cats diagnosed with CKD prior to dietary intervention.

Diet Type	n	%	Notes
Commercial supermarket diets	38	32%	Most common; non-specialized dry/wet products
Nutritionally unbalanced home-prepared diets	22	18%	Owner-prepared; variable composition
Mixed feeding (wet + dry)	26	22%	Combined commercial foods, no supplementation
Exclusive commercial (wet or dry only)	20	17%	Cats refusing alternative types
Therapeutic/prescribed diets	14	11%	Provided after previous illness
Total	120	100%	—

**Table 5:** Sensitivity analysis of CKD definition (comparison of creatinine-, SDMA-, and combined-based diagnosis).

Diagnostic Criterion	CKD Cases (n)	% of Cohort	Cohen’s κ (Agreement)
Creatinine > 1.6 mg/dL (IRIS criterion)	115	95.8%	—
SDMA > 14 µg/dL (standalone)	103	85.8%	—
Combined (creatinine and/or SDMA)	120	100%	0.91
Additional cases detected with SDMA	+5	4.2%	—

**Table 6:** Urinalysis summary by IRIS stage (USG, UPC, persistent proteinuria).

IRIS Stage	Median USG [IQR]	Median UPC [IQR]	Persistent Proteinuria (Yes/No)
I	1.025 [1.020–1.030]	0.20 [0.15–0.25]	No (borderline in 2/19)
II	1.018 [1.012–1.022]	0.28 [0.22–0.35]	Yes (7/49)
III	1.014 [1.010–1.018]	0.38 [0.30–0.46]	Yes (18/43)
IV	1.010 [1.008–1.014]	0.45 [0.39–0.55]	Yes (6/9)

**Table 7:** Blood pressure summary by IRIS stage (median BP, prevalence of hypertension, correlation with UPC).

IRIS Stage	Median BP [IQR] (mmHg)	Hypertension (≥160 mmHg) (%)	Correlation with UPC (ρ, *p*-Value)
I	135 (125–145)	10%	ρ = 0.18, *p* = 0.25
II	145 (135–155)	15%	ρ = 0.25, *p* = 0.10
III	160 (145–170)	35%	ρ = 0.39, *p* = 0.01
IV	170 (155–185)	65%	ρ = 0.42, *p* = 0.003

## Data Availability

The original contributions presented in this study are included in the article. Further inquiries can be directed to the corresponding author.
